# Supplementary material for: Effect of living arrangement on anthropometric traits in first-year university students from Canada: The GENEiUS study
Source: PLoS One. 2020 Nov 6;15(11):e0241744. doi: 10.1371/journal.pone.0241744 (PMC7647062; doi:10.1371/journal.pone.0241744)

**S3 Fig:** Distribution of waist circumference (WC) at the beginning and end of first year among students living on campus residence (n=170), in off-campus housing (n=26), and at home with family (n=48)


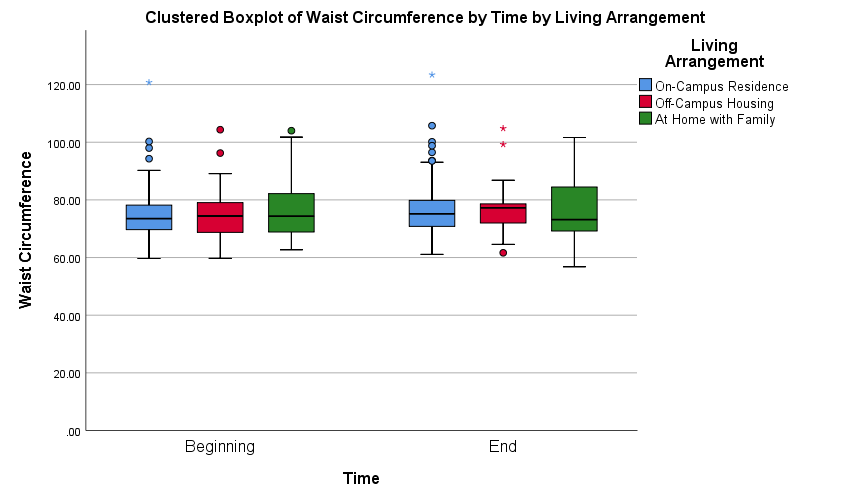

Supplement: S3 Fig — (DOCX) [file pone.0241744.s003.docx]
