# Supplementary material for: Effect of living arrangement on anthropometric traits in first-year university students from Canada: The GENEiUS study
Source: PLoS One. 2020 Nov 6;15(11):e0241744. doi: 10.1371/journal.pone.0241744 (PMC7647062; doi:10.1371/journal.pone.0241744)

**S9 Fig:** Distribution of Hip Circumference (HC) change observed over the academic year among student participants living in student residence on campus


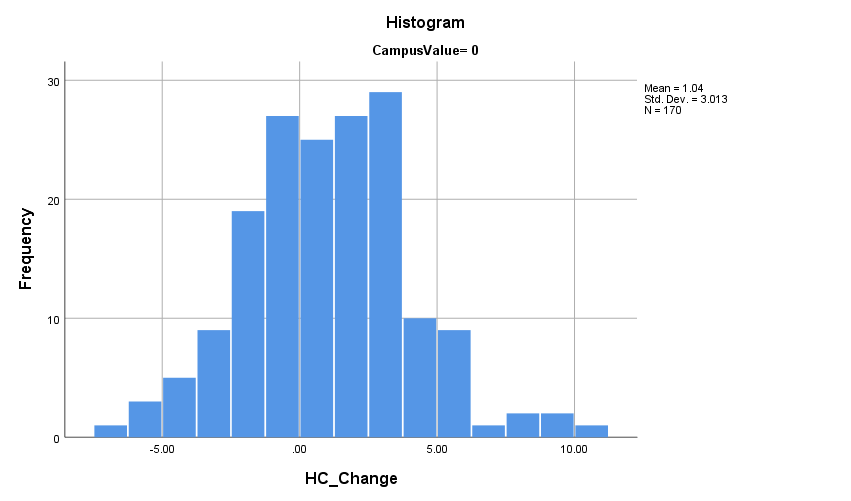

Supplement: S9 Fig — (DOCX) [file pone.0241744.s009.docx]
