# Supplementary material for: Effect of living arrangement on anthropometric traits in first-year university students from Canada: The GENEiUS study
Source: PLoS One. 2020 Nov 6;15(11):e0241744. doi: 10.1371/journal.pone.0241744 (PMC7647062; doi:10.1371/journal.pone.0241744)

**S18 Fig:** Distribution of WC change observed over the academic year among student participants living at home with family


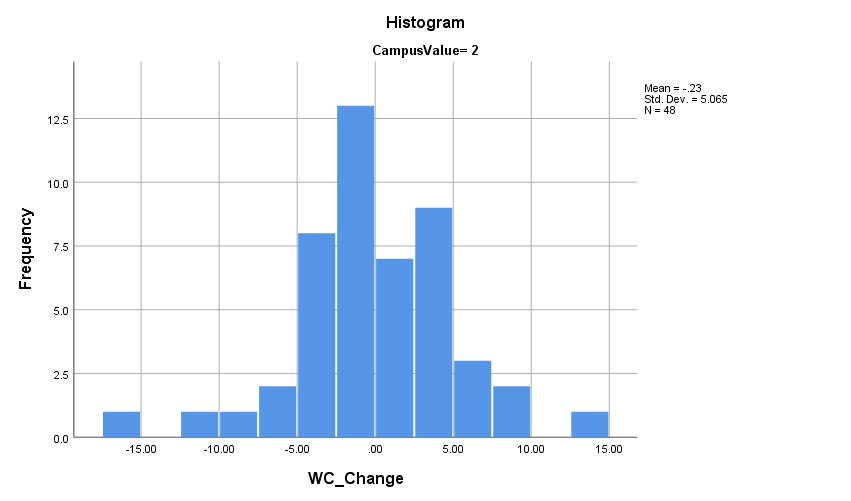

Supplement: S18 Fig — (DOCX) [file pone.0241744.s018.docx]
